# Complex marine bioturbation ecosystem engineering behaviors persisted in the wake of the end-Permian mass extinction

**DOI:** 10.1038/s41598-019-56740-0

**Published:** 2020-01-14

**Authors:** Alison T. Cribb, David J. Bottjer

**Affiliations:** Department of Earth Sciences, University of Southern California, Los Angeles, CA 90089 USA

**Keywords:** Palaeoecology, Element cycles

## Abstract

The end-Permian mass extinction was the most severe mass extinction event of the Phanerozoic and was followed by a several million-year delay in benthic ecosystem recovery. While much work has been done to understand biotic recovery in both the body and trace fossil records of the Early Triassic, almost no focus has previously been given to analyzing patterns in ecosystem engineering complexity as a result of the extinction drivers. Bioturbation is a key ecosystem engineering behavior in marine environments, as it results in changes to resource flows and the physical environment. Thus, the trace fossil record can be used to examine the effect of the end-Permian mass extinction on bioturbating ecosystem engineers. We present a dataset compiled from previously published literature to analyze burrowing ecosystem engineering behaviors through the Permian-Triassic boundary. We report two key observations: first, that there is no loss in bioturbation ecosystem engineering behaviors after the mass extinction, and second, that these persisting behaviors include deep tier, high-impact, complex ecosystem engineering. These findings suggest that while environmental conditions may have limited deeper burrowing, complex ecosystem engineering behaviors were able to persist in the Early Triassic. Furthermore, the persistence of deep tier bioirrigated three-dimensional network burrows implies that benthic biogeochemical cycling could have been maintained at pre-extinction states in some local environments, stimulating ecosystem productivity and promoting biotic recovery in the Early Triassic.

## Introduction

The end-Permian mass extinction is recognized as the most devastating mass extinction event of the Phanerozoic, resulting in an estimated loss of 81% of all marine species^1^ and a turnover of the Paleozoic evolutionary fauna to the Modern evolutionary fauna^2^. The proposed trigger of this extinction event – the eruption of the Siberian traps^3–5^ – resulted in global warming, ocean anoxia^6^, ocean acidification^7^ and habitat loss^8^. The environmental conditions following the eruption of the Siberian traps contributed to prolonged instability of biogeochemical cycles and inhabitable environments that led to a delay in global ecosystem and biotic recovery of several million years^9–11^. The nature of how ecosystems during the Early Triassic returned to stability is not currently well understood. Here, we examine the trace fossil record, an overlooked dataset to understanding biotic recovery. Compared to body fossils, trace fossils not only record the response of soft bodied organisms not easily preserved as fossils to mass extinction events^12^, but, more importantly, record the diversity and complexity of these organisms’ behaviors and, in particular, those which involve ecosystem engineering.

Ecosystem engineering refers to the behaviors of organisms which modify, create, and maintain habitable environments^13^. Ecosystem engineering can be classified as autogenic or allogenic. Autogenic ecosystem engineers modify environments by providing a physical structure for other organisms (e.g. reef-building corals), while allogenic ecosystem engineers modify environments by creating new habitats and altering resource flows by redistributing materials within the environment (e.g. vegetation-clearing goats)^13^. In the marine environment, bioturbation is the major allogenic ecosystem engineering behavior^14^ due to the resulting alteration of substrate rheology^15^, the mixing and redistribution of nutrients and sediments^16,17^, the shift in sediment redox gradients^16,18^, the creation of new habitats^17^, and the construction of new ecospace^19^. Bioturbation in marine environments typically creates a surficial mixed layer, transition layer, and historical layer^20^. The mixed layer is commonly bioturbated by mobile as well as sedentary organisms and has the highest water content. In the mixed layer, the record of bioturbation is represented by sediment mixing and a lack of preservation of identifiable trace fossils with sharp outlines. The underlying transition layer begins a number of centimeters below the sediment-water interface. The transition layer has a lower water content, as it represents the beginning of compaction, which allows for the preservation of identifiable trace fossils. Bioturbation does not typically occur in the historical layer below the transition layer. The historical layer therefore typically represents bioturbation as it ultimately appears in the stratigraphic record. In marine environments, bioturbation can be divided into various functional groups based on how bioturbators interact with the substrate within these layers^21,22^. Each behavior has its own degree of impact on the sediment chemistry, biogeochemical cycling, and benthic ecosystems^14^. Thus, the persistence or disappearance of these high-impact ecosystem engineering burrowing behaviors across the end-Permian mass extinction is key to understanding how marine ecosystems recovered in the Early Triassic.

Previous research on the trace fossil record across the Permian-Triassic boundary has primarily focused on tiering, ichnofabrics, and burrow size. Induan trace fossils tend to be simple, shallow burrows^12,23^. As early as the early Olenekian (Smithian), trace fossil assemblages exhibit recovery to pre-extinction levels of ichnodiversity, ichnofabric indices, and burrow size^24,25^. However, tiering did not return to pre-extinction depths until the beginning of the Middle Triassic^26^. Herein, we apply two ecosystem engineering framework analyses to previously published Permian-Triassic trace fossil datasets to understand the patterns of ecosystem engineering behaviors across the mass extinction boundary and how these behaviors may have influenced ecosystem recovery in the Early Triassic.

## Materials and Methods

### Ecosystem engineering behavior and trace fossil data collection

The objective of this research is to use the Permian-Triassic trace fossil record to compile and analyze ecosystem engineering behaviors and their impacts on the benthic environment. Trace fossil occurrences were compiled from previously published literature and included the entire Permian through the first stage of the Middle Triassic (Asselian through the Anisian, 299–237 Ma^27^). Only trace fossils which could be confidently assigned to a tier – surficial, semi-infaunal, shallow, intermediate, or deep^28–30^ – were added to the dataset. Tiering is a fundamental part of the ecosystem engineering analyses used in this research, and thus trace fossils without a precise description of penetration depth or tiering cannot be accurately analyzed in terms of ecosystem engineering behavior and the effect it may have had on the benthic environment. Each ichnogenus was counted once per tier because the presence or absence of each ecosystem engineering behavior represents the necessary data to investigate the effect of the extinction on each behavior and the roll that each behavior may have played in the recovery from the extinction. Data were also limited to shallow marine trace fossil occurrences as they generally provide the richest trace fossil record^26^. Due to data filtering based on the lack of precise tiering descriptions, Roadian and Capitanian data are absent. Ultimately, 164 unique ichnogenera were entered into the database given the parameters of the study (confident tiering assignment, one occurrence per tier, one occurrence per time period, and shallow marine). These data were analyzed using two frameworks: the ecosystem engineering occupation cube method^22^ and the ecosystem engineering impact (EEI) values method^14,31^. Trace fossils and their categorical assignments for both ecosystem engineering analyses were grouped into stages based on stratigraphic descriptions in the primary literature.

### Ecosystem engineering occupation cubes

The ecosystem engineering occupation cube method^22^ assigns ‘cube spaces’ to ichnogenera according to tiering, sediment interaction^32^, and sediment modification^21,22^. For tiering, literature descriptions were used to classify each trace fossil entry as surficial, semi-infaunal (0–0.5 cm), shallow (0.5–6 cm), intermediate (6–12 cm), or deep (>12 cm)^28–30^. Surficial, semi-infaunal, and shallow tier bioturbating organisms commonly occupy the mixed layer, while bioturbators occupying intermediate or deep tiers occupy the transition layer. Each ichnogenus was assigned one of four sediment interaction classifications: intrusion, compression, backfilling, and excavation^32^. Intrusion describes the displacement of the sediment as the animal burrows and sediment closes up behind it; compression describes the movement and compaction around the burrowing animal; backfilling describes the backward passage of sediment either around or through the burrower; and excavation describes the active loosening and transportation of sediment from one point along the burrow path to another^32^. Each ichnogenus was also assigned one of four sediment modification classifications: biodiffusion, gallery biodiffusion, conveyor, and regenerator^21^. Biodiffusion involves the movement of sediment particles over short distances; gallery biodiffusion involves the redistribution of sediment particles from one part of the sediment profile to another; conveying involves transporting sediment particles across and within tiers; and regenerating involves moving sediment up to the surface from below the sediment-water interface^21^. When possible, each ichnogenus was given sediment modification and sediment interaction assignments based on previous descriptions^22^. For ichnogenera which had not been previously described in this framework, the original literature which described the ichnogenera was consulted for the assignments. From these tiering, sediment modification, and sediment interaction categories, each ichnogenus was given an occupied cube which represents a certain ecosystem engineering behavior within a given tiering depth. For each of the time intervals across the Permian-Triassic boundary, the number of occupied cubes is summed to represent the total number of ecosystem engineering behaviors present.

### Ecosystem engineering impact values

The ecosystem engineering impact (EEI) value method^14^ rank-order scores trace fossils on the basis of tiering, bioturbation behavior functional group^21^, and bioirrigation potential^14^. We have modified this method to use the same tiering categories used in the ecosystem engineering occupation cube scheme in order to make the two frameworks more comparable. Potential functional group, which describes how the burrowing organism modifies the substrate, scores trace fossils as 1 = epifaunal locomotion, 2 = surficial modification, 3 = biodiffusion, 4 = regeneration, 5 = downward conveying, 6 = upward conveying, or 7 = gallery biodiffusion. Biodiffusion, regeneration, and gallery biodiffusion are the same descriptions as those in the ecosystem engineering occupation cube framework^22^. Epifaunal locomotion describes a surficial animal’s movement which does not penetrate the sediment-water interface. Surficial modification describes burrowing which moves particles over short distances only within 2 cm of the sediment-water interface. Conveying is divided into downward conveying, which describes head-down orientation movement, and upward-conveying, which describes movement of an organism with its head at or close to the surface^21^. Bioirrigation potential, which describes the likelihood that the burrow was flushed with sediment, is scored as 1 = improbable, 2 = probable, and 3 = possible. Many ichnogenera can occupy a range in values for each category, so they are given a summated EEI range. The final EEI value range for an ichnogenus is calculated by summing the minimum scores and the maximum scores. Trace fossils which represent simple, low-impact ecosystem engineering have scores lower in range and value, and trace fossils which represent complex, high-impact ecosystem engineering have scores higher in range and value^14^.

## Results

### Patterns in burrow tiering

**Figure 1:**
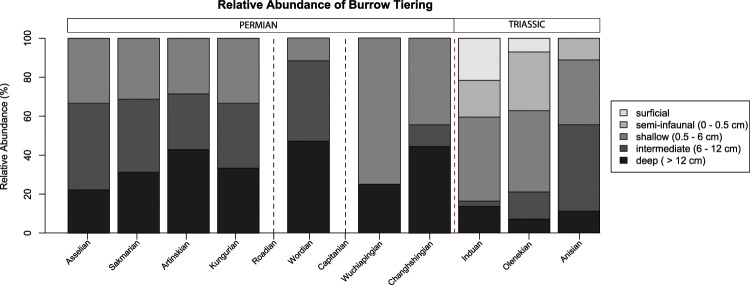
Relative abundance of burrow tiering across the Permian-Triassic. Trace fossils for the Roadian and Capitanian are absent (see details in Materials and Methods), represented by vertical gray dashed lines. end-Permian mass extinction is represented by the vertical red dashed line. Percentages for trace fossils representing each tier in a given time interval are in Supplemental Material, S2.

The Permian primarily consists of deep and intermediate tier burrows, with a smaller component of shallow tier burrows and no reported semi-infaunal and surficial burrows (Fig. 1). The relative abundance of deep tier burrows is stable until it decreases by about a third from the Wordian to the Wuchiapingian and increases again in the Changhsingian. Intermediate tier burrows follow a similar pattern but disappear in the Wuchiapingian and are only a tenth of the trace fossils in the Changhisingian. Abundance of shallow tier burrows is stable from the Asselian to the Kungurian, decreases in the Wordian, increases during the Wuchiapingian, and decreases in the Changhsingian. Surficial and semi-infaunal tier burrows were not reported in the Permian.

The Triassic marks a shift to a majority of shallow and semi-infaunal burrows. Surficial trace fossils are only present in the Induan and Olenekian. Semi-infaunal tier burrows increase in abundance from the Induan to the Olenekian and decrease in the Anisian (Fig. 1). Shallow tier burrows represent nearly half of the trace fossils in the Induan and continue to make up at least 40% of all trace fossils at each stage throughout the Triassic. Intermediate and deep burrows combined represent less than a fifth of total trace fossils in the Induan and Olenekian and increase to half of all trace fossils in the Anisian.

### Ecosystem engineering occupation cubes

**Figure 2:**
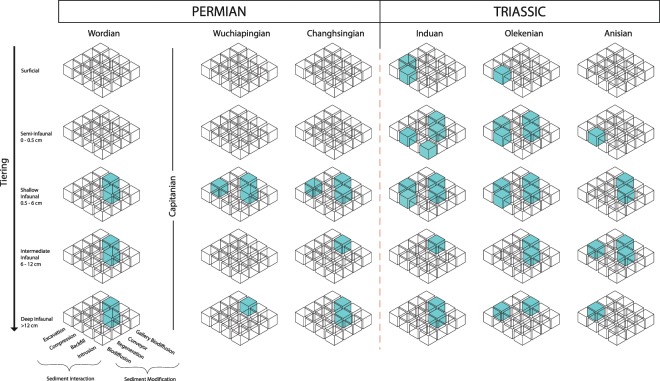
Ecosystem engineering occupation cubes^22^. Teal shaded cubes indicate ecosystem engineering behavior which is represented by a present ichnogenus in a given tiering depth. The Permian-Triassic mass extinction event is represented by the vertical red dashed line. Number of ichnogenera which occupy each cube are given in Supplemental Material, S3. Occupied cubes for the Asselian to the Anisian given in Supplemental Material, S4.

During the Permian, there are four occupied cubes in the Asselian, six in the Sakmarian, six in the Artinskian, five in the Kungurian, six in the Wordian, four in the Wuchiapingian, and six in the Changhsingian. During the Triassic, there are thirteen occupied cubes in the Induan and in the Olenekian (the maximum at all time intervals), and seven in the Anisian (Fig. 2; Supplemental Material, S3). Five behaviors represented by interaction-modification combinations comprise all cubes across the Permian-Triassic: regenerator-excavation, compression-biodiffusion, intrusion-biodiffusion, backfill-conveyor, and gallery biodiffusion-compression. Gallery biodiffusion-compression is the most common ecosystem engineering behavior (Fig. 2; Supplemental Materials, S1; S3).

### Ecosystem engineering impact values

From the Asselian to the Kungurian, ecosystem engineering impact is high in both value and range at EEI = 7–14 (Fig. 2). In the Wordian, EEI values increase in range to EEI = 5–14. EEI values remain high but decrease in range to EEI = 7–14 for the Wuchiapingian and Changhsingian. In the Induan, EEI values increase to EEI = 3–14 and remain consistent into the Olenekian. EEI values decrease in range to EEI = 4–14 in the Anisian.

## Discussion

### Persistence of high-impact ecosystem engineering behaviors

The ecosystem engineering occupation cubes reveal that there are no ecosystem engineering bioturbation behaviors (sediment interaction-sediment modification combination) lost in the aftermath of the end-Permian mass extinction (Fig. 2). We note that some complex trace fossils, such as *Zoophycos*, do disappear during the Early Triassic, but other trace fossils representing the same ecosystem engineering behaviors continue to persist. The three ecosystem engineering behaviors present in the Permian – compression-gallery biodiffusion, backfill conveyor, and compression-biodiffusion – are still present even in the Induan. These results are in agreement with previous observations that, despite the high extinction rates and selectivity associated with the end-Permian mass extinction, there was little change in functional group diversity between pre- and post-extinction benthic ecosystems^33,34^.

**Figure 3:**
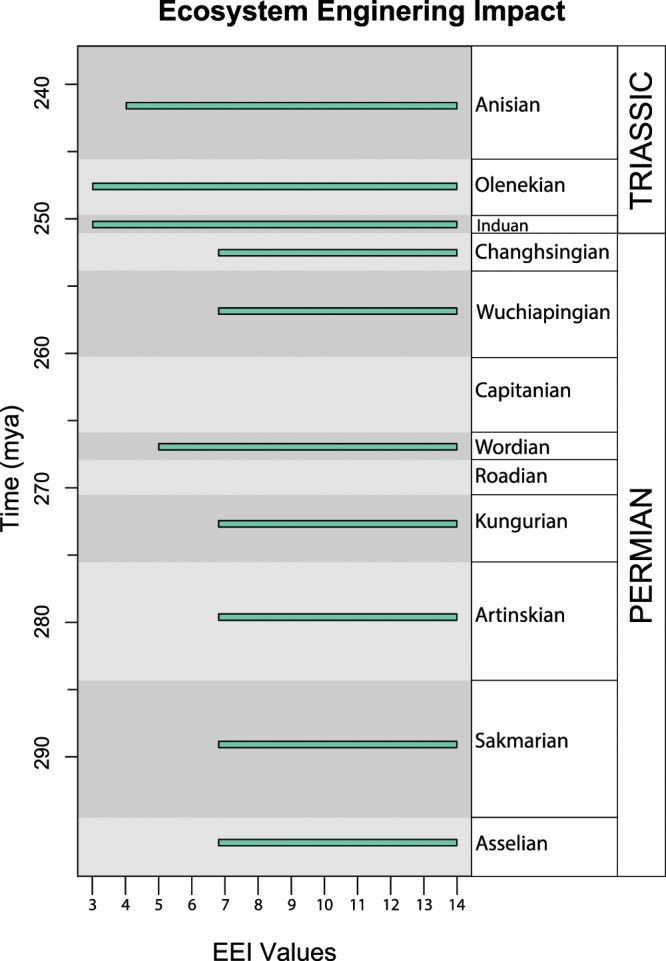
Ecosystem engineering impact (EEI) values and ranges^14^. Teal bars represent the range between the minimum and maximum EEI value scores for all the ichnogenera in a given time interval. Mass extinction event occurs between the Changhsingian and the Induan.

Notably, gallery biodiffusion, the highest impact sediment modification functional group^14^, is present even in the deepest tiers in the Induan and Olenekian following the mass extinction event due to the persistence of trace fossils such as *Skolithos*, *Diplocraterion*, and *Thalassinoides*^22^ (Supplemental Material, S1). Moreover, the continuation of high EEI values through the Early Triassic reveals that ecosystem engineering remains both complex and high-impact across the Permian-Triassic boundary (Fig. 3). Any effect of the mass extinction on ecosystem engineering is evident only in a decrease in minimum EEI values from the Changhsingian to the Induan (Fig. 3), but this decrease in EEI values without a contraction in range during the Triassic reflects presence of semi-infaunal and surficial trace fossils rather than the loss of high-impact ecosystem engineering (Figs. 1 and 2). The persistence of all ecosystem engineering behaviors likely reflects either behavioral redundancy in bioturbating ecosystem engineers or that new Early Triassic bioturbators rapidly refilled empty roles of the extinct Permian ecosystem engineers^34^.

### Collapse of bioturbation depth in the Early Triassic

Although no loss in ecosystem engineering behaviors is evident, the data do reveal a collapse in bioturbation depth associated with the extinction (Fig. 1). It is also evident that there are fewer ichnogenera that represent high-impact ecosystem engineering behaviors present in the deep and intermediate tiers during the Early Triassic than during the Permian (Fig. 3; Supplemental Materials S2, S3). Figure 2, ecosystem engineering occupation cubes for example, only three ichnogenera in the Induan occupy the deep compression-gallery biodiffusion cube space, whereas five ichnogenera occupy the same cube space during the Wordian. The lack of surficial and semi-infaunal trace fossils during the Permian is best explained by the existence of a well-developed mixed layer^35^, while the predominance of shallow tier burrows and loss of deep tiering burrows in the Triassic implies the loss of deep sedimentary mixing in shallow marine environments (Fig. 1). This has been observed in previous research at a variety of temporal and spatial scales^12,26,36,37^.

What caused the loss of deep tier burrows remains unclear. On the one hand, the Early Triassic has been widely associated with widespread marine anoxia^6,38–40^. In modern environments and controlled experiments, some animal burrows become shallower in hypoxic settings^39,41,42^. Thus, the collapse of deep sedimentary bioturbation and lack of recovery by the end of the Early Triassic could be interpreted as the persistence of low-oxygen conditions in shallow marine environments. The persistence of some high-impact ecosystem engineering behaviors (e.g. gallery biodiffusion) in the deep tiers in the Early Triassic, however, most likely suggests that across all localities in the dataset, environmental stress limited burrowing organisms to shallower depths, but some local conditions may have permitted deeper burrowing. This explanation is in agreement with the differences in functional group extinction across the Permian-Triassic boundary reported in different depositional environments and paleolatitudes^33^. Furthermore, deep tier trace fossils tend to occur in the shallowest marine environments, where coarse sediment allows for easier oxygen diffusion deeper into the substrate from the sediment-water interface. These shallow facies are not present in trace-fossil bearing Olenekian sections, which likely explains the lower proportion of deep tier trace fossils after the Induan. Finally, we note that the proxies by which marine anoxia are determined may be locally controlled by the intensity of bioturbation, as bioirrigation promotes oxygen penetration into the sediment^16,26^. The geochemical signal of anoxia may, therefore, be amplified by the lack of deeper bioturbation.

### Implications for ecosystem recovery

The persistence of high-impact ecosystem engineering behaviors and loss of deep bioturbation across the end-Permian mass extinction both likely affected benthic biogeochemical cycling during the Early Triassic. Bioturbation exerts a control on the rate of nutrient cycling in marine ecosystems^43,44^. Globally, a cessation of deep tier bioturbation in general may have increased nutrient burial, as nutrients that reach the seafloor would not have been as likely to be reworked by conveyors and regenerators and resuspended back into the water column. Additionally, a shallowing of the mixed and transition layers would have caused the redox profile discontinuity (RPD) to migrate upwards towards the sediment-water interface^16,42^, which may have affected redox-sensitive nutrient flux and benthic microbial processes^45^. However, in some local environments, deep high-impact ecosystem engineering behaviors would have maintained benthic nutrient flux and the RPD depth by transporting nutrients throughout the sediment and promoting nutrient flow to microbial communities^46^. More specifically, gallery biodiffusion (the highest impact bioturbation functional group^14^), vastly increases the surface area for solute exchange and active biogeochemical reaction sites^47–49^. This means that these complex bioturbation behaviors are a critical aspect of benthic nitrogen cycling, for example, as nitrification-denitrification processes require an oxic-anoxic interface^50^. Increased bioirrigation through gallery network burrows thus can result in increased areas of stimulated nitrification^51^ and decrease the flux of inorganic nitrogen to the water column^52^. Bioirrigation may also influence benthic phosphate cycling, as phosphate adsorbs to positively charged clay particles in oxic conditions^49^. Increased bioturbation intensity by animals which create bioirrigated gallery networks has also been found to cause increased abundance of microbial communities which are unique to the burrow, either due to increased oxygen^45^ or organic matter in the burrow wall^53^. Thus, as these high-impact ecosystem engineering behaviors and burrow size returned to pre-extinction levels within the intermediate and deep tiers, progressively more complex bioturbation would have allowed benthic biogeochemical cycling to return to its pre-extinction state.

Precisely how these bioturbation-induced changes in biogeochemical cycling may have influenced biotic recovery of benthic macrofauna is not entirely resolved, and research investigating the links between these benthic processes remains limited. However, a number of studies of modern benthic ecology have linked habitat quality and ecosystem functioning to oxygenated and bioturbated substrates^52,54,55^. An increased supply of nutrients to microbes due to complex bioturbation behaviors would have stimulated marine productivity, which may have maintained or increased the diversity of life^27,40,55,56^ in the wake of the end-Permian mass extinction. However, it is still implausible that ecosystems would have fully recovered until larger, deep tier bioturbators regained pre-extinction levels on a global scale^33^. Although, there is still potential for high-impact ecosystem engineering behaviors, most notably gallery biodiffusion, to maintain habitable environments during biotic crises and mass extinctions, particularly in environments where deep tier burrows persisted in the Early Triassic.

## Conclusions

The data presented here show two key observations: (1) there is no loss in ecosystem engineering bioturbation behavior after the end-Permian mass extinction, and (2) the highest-impact ecosystem engineering behaviors (deep tier gallery biodiffusion) persisted in the Early Triassic. There is a pronounced shift from deep and intermediate tier burrows in the Permian to shallow and semi-infaunal tier burrows in the Early Triassic, likely related to significant loss of mixed layer development and shallowing of the transition layer. However, ecosystem engineering complexity does not seem to be majorly affected, as highly effective sediment mixing behaviors were still present throughout the entire Early Triassic. These two key observations suggest that although shallow marine environments may have still been inhospitable during the Early Triassic, generally causing bioturbating organisms to be limited to the shallow tier, some local environmental conditions were favorable enough to bioturbators to reach the deep sediment tier with rather complex bioturbation behaviors. The persistence of complex engineering behaviors across the most devastating mass extinction in Earth history suggests that there was still potential for these organisms to maintain habitable benthic environments by increasing nutrient flow throughout the sediment profile and to the surface.

## Supplementary information

Supplementary Information.

Supplementary Information2.

## References

[1] Stanley SM (2016). Estimates of the magnitudes of major mass extinctions in earth history. PNAS.

[2] Sepkoski JJ (1981). A factor analytic description of the Phanerozoic marine fossil record. Paleobiology.

[3] Campbell I, Czamanske G, Fedoernko V, Hil R, Staphanov V (1992). Synchronism of the Siberian trap and the Permian-Triassic boundary. Science.

[4] Reichow MK (2009). The timing and extent of the eruption of the Siberian Traps large igneous province: implications for the end-Permian environmental crisis. Earth and Planetary Science Letters.

[5] Burgess SD, Bowring S, Shen S (2014). High-precision timeline for Earth’s most severe extinction. PNAS.

[6] Song, H. *et al*. Anoxia/high temperature double whammy during the Permian-Triassic marine crisis and its aftermath. *Scientific Reports* **4123** (2014) 10.1038/srep04132 PMC3928575 24549265

[7] Clarkson M (2015). Ocean acidification and the Permo-Triassic mass extinction. Science.

[8] Erwin D (1994). The Permo-Triassic extinction. Nature.

[9] Payne JL (2004). Large perturbations of the carbon cycle during recovery from the end-Permian extinction. Science.

[10] Lehrmann DJ (2006). Timing of recovery from the end-Permian extinction: geochronologic and biostratigraphic constrains from south China. Geology.

[11] Bottjer DJ, Clapham M, Fraiser M, Powers C (2008). Understanding the mechanisms for the end-Permian mass extinction and the protracted Early Triassic aftermath and recovery. GSA Today.

[12] Twitchett RJ, Barras CG (2004). Trace fossils in the aftermath of mass extinction events. Geological Society, London, Special Publiations.

[13] Jones, C. G., Lawton, J. H. & Shachak, M. Organisms as ecosystem engineers. In *Ecosystem Management* (ed. Samson, F. B. & Knopf, F. L.) 130–147 (Springer 1994).

[14] Herringshaw LG, Callow RHT, McIlroy D (2017). Engineering the Cambrian explosion: the earliest bioturbators as ecosystem engineers. Geological Society, London, Special Publications.

[15] De Decekere EMGT, Tolhurts TJ, De Brouwer JFC (2001). Destabilization of cohesive intertidal sediments by infauna. Coastal and Shelf Science.

[16] Aller, R. The effects of macrobenthos on chemical properties of marine sediment and overlying water. In *Animal-Sediment Relations* (ed. McCall, P. & Tevesz, M. L.) 53–102 (Springer 1982).

[17] McIlroy D, Logan GA (1999). The impact of bioturbation on infaunal ecology and evolution during the Proterozoic-Cambrian transition. Palaios.

[18] Erwin D, Tweedt S (2012). Ecological drivers of the Ediacaran-Cambrian diversification of Metazoa. Evolutionary Ecology.

[19] Erwin D (2008). Macroevolution of ecosystem engineering, nice construction and diversity. Trends in Ecology & Evolution.

[20] Savrda Charles E. (2007). Trace Fossils and Marine Benthic Oxygenation. Trace Fossils.

[21] Solan M. & Wigham, B. D. Biogenic particle reworking and bacterial-invertebrate interactions in marine sediments. In *Interactions between Macro- and Microorganisms in Marine Sediments* (ed. Kristensen, E.) 105–124 (American Geophysical Union 2004).

[22] Minter, N. J. *et al*. Early bursts of diversification defined the faunal colonization of land. *Nature Ecology & Evolution* **1** (2017).

[23] Pruss SB, Bottjer DJ (2004). Early Triassic trace fossils of the western United States and their implications for prolonged environmental stress from the end-Permian mass extinction. Palaios.

[24] Chen Z, Fraiser M, Bolton C (2012). Early Triassic trace fossils from Gondwana Interior Sea: Implications for ecosystem recovery following the end-Permian mass extinction in south high-latitude region. Gondwana Research.

[25] Hofmann R, Goudemand R, Wasmer M, Bucher H, Hautmann M (2011). New trace fossil evidence for an early recovery signal in the aftermath of the end-Permian mass extinction. Palaeogeography, Palaeoclimatology, Palaeoecology.

[26] Hofmann R, Buatois LA, MacNaughton RB, Mángano MG (2015). Loss of the sedimentary mixed layer as a result of the end-Permian mass extinction. Palaeogeography, Palaeoclimatology, Palaeoecology.

[27] Gradstein FM, Ogg JG, van Kranedonk M (2008). On the geologic time scale. Newsletters on Stratigraphy.

[28] Ausich WI, Bottjer DJ (1982). Tiering in suspension feeding communities on soft substrata throughout the Phanerozoic. Science.

[29] Bottjer DJ, Ausich WI (1986). Phanerozoic development of tiering in soft substrata suspension-feeding communities. Paleobiology.

[30] Mángano MG, Buatois LA (2014). Decoupling of body-plan diversification and ecological structuring during the Ediacaran-Cambrian transition: evolutionary and geobiological feedbacks. Proceedings of the Royal Society B.

[31] Cribb AT (2019). Increase in metazoan ecosystem engineering prior to the Ediacaran-Cambrian boundary in the Nama Group, Namibia. Royal Society Open Science.

[32] Buatois, L. A. & Mángano, M. G. *Ichnology: organism-substrate interactions in space and time*. (Cambridge University Press, 2011).

[33] Foster WJ, Twitchett RJ (2014). Functional diversity of marine ecosystems after the Late Permian mass extinction event. Nature Geosciences.

[34] Dineen AA, Roopnarine PD, Fraiser ML (2019). Ecological continuity and transformation after the Permo-Triassic mass extinction in northeastern Panthalassa. Biology Letters.

[35] Buatois LA, Mángano GM (2013). Ichnodiversity and ichnodisparity: significance and caveats. Lethaia.

[36] Ausich, W. I. & Bottjer, D. J. Sessile invertebrates. In *Palaeobiology II* (ed. Briggs, D. E. G. & Crowther, P. R.) 384–386 (Blackwell Science Ltd, 2001).

[37] Twitchett RJ (2006). The palaeoclimatology, palaeoecology, and palaeoenvironmental analysis of mass exintction events. Palaeogeography, Palaeoclimatology, Palaeoecology.

[38] Wignall PB, Twitchett RJ (1996). Oceanic anoxia and the end Permian mass extinction. Science.

[39] Savrda CE, Bottjer DJ (1986). Trace-fossil model for reconstruction of paleo-oxygenation in bottom waters. Geology.

[40] Bond DP, Wignall PB (2010). Pyrite framboid study of marine Permian-Triassic boundary sections: a complex anoxic event and its relationship to contemporaneous mass extinctions. GSA Bulliten.

[41] Diaz RJ, Rosenberg R (1995). Marine benthic hypoxia: A review of its ecological effects and the behavioural responses of benthic macrofauna. Oceanography and marine biology. An annual review.

[42] Weissberger EJ, Coiro LL, Davey EW (2009). Effects of hypoxia on sediment redox profiles. Journal of Experimental Marine Biology and Ecology.

[43] Biles CL, Paterson DM, Ford RB, Solan M, Rafaelli DG (2002). Bioturbation, ecosystem functioning, and community structure. Hydrology and Earth Systems Sciences Discussions.

[44] Laverock B, Gilbert JA, Tait K, Osborn AM, Widdicombe S (2011). Bioturbation: impact on the marine nitrogen cycle. Biochemical Society Transactions.

[45] Bertics VJ, Ziebis W (2009). Biodiversity of benthic microbial communites in bioturbated coastal sediments is controlled by geochemical microniches. The International Society of Microbial Ecology Journal.

[46] Mermollid-Blondin F, Rosenberg F (2006). Ecosystem engineering: the impact of bioturbation on biogeochemical processes in marine and freshwater benthic habitats. Aquatic Science.

[47] Aller RC (1983). The importance of the diffusive permeability of animal burrow linings in determining marine sediment chemistry. Journal of Marine Research.

[48] Huettel M, Berg P, Kostka JE (2013). Benthic exchange and biogeochemical cycling in permeable sediments. Annual Review of Marine Science.

[49] Lohrer AM, Thrush SF, Gibbs MM (2004). Bioturbators enhance ecosystem function through complex biogeochemical interactions. Nature.

[50] Jenkins MC, Kemp WM (1984). The coupling of nitrification and denitrification in two estuarine sediments. Limnology and Oceanography.

[51] Huettel M, Ziebis W, Forster S, Luther GW (1998). Advective transport affecting metal and nutrient distributions and interfacial fluxes in permeable shelf sediments. Geochemica Cosmochemica Acta.

[52] Nilsson HC, Rosenberg R (2000). Succession in marine benthic habitats and fauna in response to oxygen deficiency: analysed by sediment profile-imaging and by grab samples. Marine Ecology Progress Series.

[53] Papaspyrou S, Gregersen T, Kristensen E, Christense B, Fox RP (2006). Microbial reaction rates and bacterial communities in sediment surrounding burrows of two nereidid polychaetes (*Nereis diversicolor* and *N. verins*). Marine Biology.

[54] Teal LR, Parker ER, Solan M (2010). Sediment mixed layer as a proxy for benthic ecosystem process and function. Marine Ecology Progress Series.

[55] Nilsson HC, Rosenberg R (1997). Benthic habitati quality assessment of an oxygen stressed fjord by surface sediment profile images. Journal of Marine Systems.

[56] Martin RE, Quigg A, Podkovyrov V (2008). Marine biodiversification in response to evolving phytoplankton stoichiometry. Palaeogeography, Palaeoclimatology, Palaeoecology.

