# Supplementary material for: Complex marine bioturbation ecosystem engineering behaviors persisted in the wake of the end-Permian mass extinction
Source: Sci Rep. 2020 Jan 14;10:203. doi: 10.1038/s41598-019-56740-0 (PMC6959249; doi:10.1038/s41598-019-56740-0)

Supplemental material for 'Complex marine bioturbation ecosystem engineering behaviors persisted in the wake of the end-Permian mass extinction'

Alison T. Cribb, David J. Bottjer

S2. Burrow Tiering Percentages

Stage/Substage	% surficial	% semi-infaunal	% shallow	% intermediate	% deep	Sample size (n)
Asselian	0	0	33.3	44.4	22.2	9
Sakmarian	0	0	31.3	37.5	31.2	16
Artinskian	0	0	28.6	28.6	42.8	14
Kungurian	0	0	33.3	33.3	33.3	6
Wordian	0	0	11.8	41.2	47.1	17
Wuchiapingian	0	0	75.0	0	25.0	4
Changhsingian	0	0	40.0	10.0	50.0	9
Induan	21.6	18.9	43.2	2.7	13.5	37
Olenekian	7.0	30.1	41.9	14.0	7.0	43
Anisian	0	11.1	33.3	44.4	11.1	9

S3. Asselian to Anisian Ecosystem Engineering Cube Assignments

The following table shows the number of ichnogenera in each time interval that are present for each ecosystem engineering cube space, denoted by the ecosystem engineering behavior tier + interaction + modification combinations (Minter et al., 2017). Highlighted, bolded numbers correspond to the number of ichnogenera at each stage that correspond to that particular ecosystem engineering behavior. Ichnogenera occurrences are counted only once in each tier for each time interval (see Methods for more details on dataset creation).

**Impacts upon sediment**	Asselian	Sakmarian	Artinskian	Kungurian	Wordian	Wuchianpingian	Changhsingian	Induan	Olenekian	Anisian
Surficial + Excavation + Regenerator	0	0	0	0	0	0	0	**2**	0	0
Surficial + Compression + Biodiffusion	0	0	0	0	0	0	0	**6**	**3**	0
Semi-infaunal + Backfill + Conveyor	0	0	0	0	0	0	0	**1**	**3**	0
Semi-infaunal + Compression + Gallery Biodiffusion	0	0	0	0	0	0	0	**1**	**3**	0
Semi-infaunal + Intrusion + Biodiffusion	0	0	0	0	0	0	0	**1**	0	0
Semi-Infaunal + Excavation + Regenerator	0	0	0	0	0	0	0	0	**1**	0
Semi-infaunal + Compression + Biodiffusion	0	0	0	0	0	0	0	**4**	**6**	**1**
Shallow + Compression + Biodiffusion	0	0	0	0	0	0	0	**1**	**1**	0
Shallow + Compression + Gallery Biodiffusion	0	**1**	**1**	**1**	**1**	**1**	**1**	**7**	**10**	**2**
Shallow + Backfill + Conveyor	**3**	**4**	**3**	**1**	**1**	**1**	**2**	**5**	**5**	**2**
Shallow + Excavation + Regenerator	0	0	0	0	0	**1**	**1**	**3**	**2**	0
Intermediate + Compression + Gallery Biodiffusion	**3**	**3**	**2**	0	**2**	0	**1**	**1**	**4**	**2**
Intermediate + Backfill + Conveyor	0	**3**	**2**	**2**	**5**	0	0	0	**2**	**1**
Intermediate + Excavation + Regenerator	**1**	0	0	0	0	0	0	0	0	**1**
Deep + Compression + Gallery Biodiffusion	**2**	**3**	**5**	**1**	**5**	**1**	**3**	**3**	**2**	0
Deep + Backfill + Conveyor	0	**2**	**1**	**1**	**3**	0	**1**	**2**	0	0
Deep + Excavation + Regenerator	0	0	0	0	0	0	0	0	**1**	**1**

References

Minter, N. J., Buatois, L. A., Mángano, M. G., Davies, N. S., Gibling, M. R., MacNaughton, R., & Labandeira, C. C. Early bursts of diversification defined the faunal colonization of land. *Nature Ecology & Evolution* **1** (2017).

S4. Asselian to Anisian Ecosystem Engineering Cubes

The following figure shows the occupied ecosystem engineering cubes for the entire dataset.

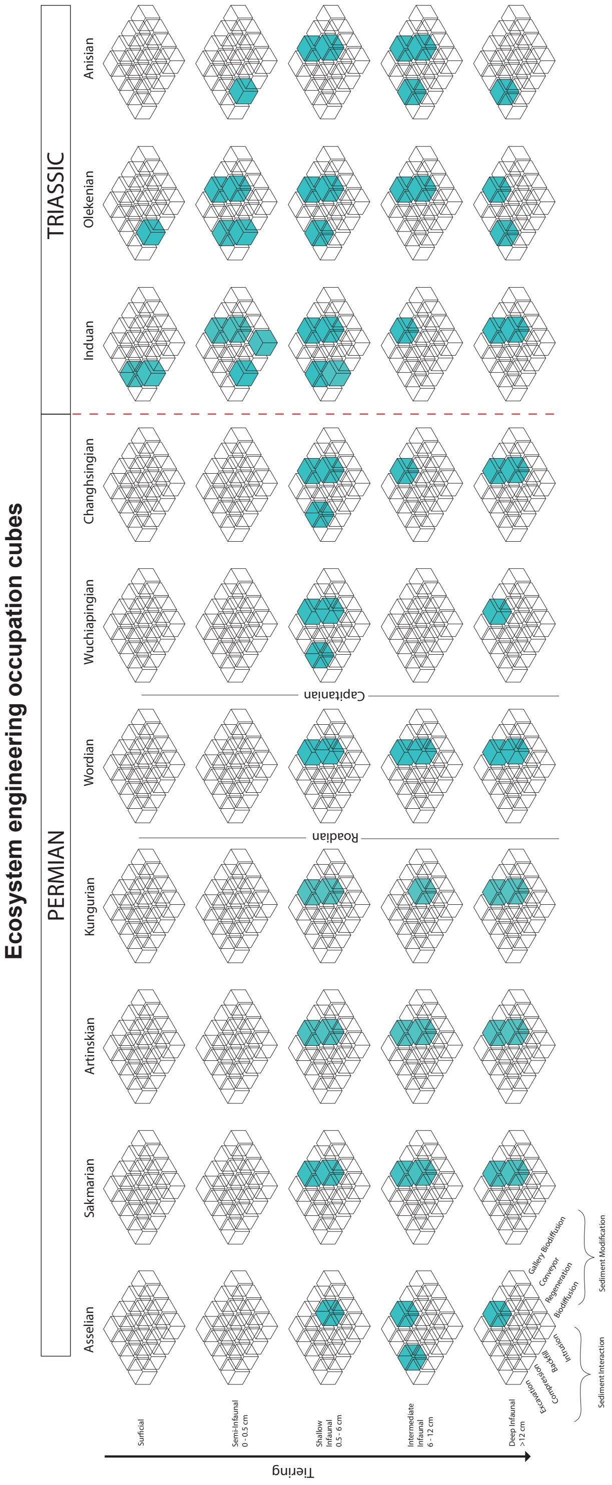

Supplement: Supplementary file 2 — Supplementary Information2. [file 41598_2019_56740_MOESM2_ESM.pdf]
